# What is the Accuracy of Nuclear Imaging in the Assessment of Periprosthetic Knee Infection? A Meta-analysis

**DOI:** 10.1007/s11999-016-5218-0

**Published:** 2017-01-03

**Authors:** Steven J. Verberne, Remko J. A. Sonnega, Olivier P. P. Temmerman, Pieter G. Raijmakers

**Affiliations:** 1 Department of Orthopaedics, Noordwest Ziekenhuisgroep, Wilhelminalaan 12, 1815 JD Alkmaar, NWZ The Netherlands; 2 The Centre for Orthopaedic Research Alkmaar (CORAL), Noordwest Ziekenhuisgroep, Alkmaar, The Netherlands; 3 Department of Radiology & Nuclear Medicine, VU University Medical Centre, Amsterdam, The Netherlands

## Abstract

**Background:**

In the assessment of possible periprosthetic knee infection, various imaging modalities are used without consensus regarding the most accurate technique.

**Questions/Purposes:**

To perform a meta-analysis to compare the accuracy of various applied imaging modalities in the assessment of periprosthetic knee infection.

**Methods:**

A systematic review and meta-analysis was conducted with a comprehensive search of MEDLINE and Embase^®^ in accordance with the PRISMA and Quality Assessment of Diagnostic Accuracy Studies (QUADAS-2) recommendations to identify clinical studies in which periprosthetic knee infection was investigated with different imaging modalities. The sensitivity and specificity of each imaging technique were determined and compared with the results of microbiologic and histologic analyses, intraoperative findings, and clinical followup of more than 6 months. A total of 23 studies, published between 1990 and 2015, were included for meta-analysis, representing 1027 diagnostic images of symptomatic knee prostheses. Quality of the included studies showed low concerns regarding external validity, whereas internal validity indicated more concerns regarding the risk of bias. The most important concerns were found in the lack of uniform criteria for the diagnosis of a periprosthetic infection and the flow and timing of the included studies. Differences among techniques were tested at a probability less than 0.05 level. Where there was slight overlap of confidence intervals for two means, it is possible for the point estimates to be statistically different from one another at a probability less than 0.05. The z-test was used to statistically analyze differences in these situations.

**Results:**

Bone scintigraphy was less specific than all other modalities tested (56%; 95% CI, 0.47–0.64; p < 0.001), and leukocyte scintigraphy (77%; 95% CI, 0.69–0.85) was less specific than antigranulocyte scintigraphy (95%; 95% CI, 0.88–0.98; p < 0.001) or combined leukocyte and bone marrow scintigraphy (93%; 95% CI, 0.86–0.97; p < 0.001). Fluorodeoxyglucose positron emission tomography (FDG-PET) (84%; 95% CI, 0.76–0.90) was more specific than bone scintigraphy (56%; 95% CI, 0.47–0.64; p < 0.001), and less specific than antigranulocyte scintigraphy (95%; 95% CI, 0.88–0.98; p = 0.02) and combined leukocyte and bone marrow scintigraphy (93%; 95% CI, 0.86–0.97; p < 0.001). Leukocyte scintigraphy (88%; 95% CI, 0.81–0.93; p = 0.01) and antigranulocyte scintigraphy (90%; 95% CI, 0.78–0.96; p = 0.02) were more sensitive than FGD-PET (70%; 95% CI, 0.56–0.81). However, because of broad overlapping of confidence intervals, no differences in sensitivity were observed among the other modalities, including combined bone scintigraphy (93%; 95% CI, 0.85–0.98) or combined leukocyte and bone marrow scintigraphy (80%; 95% CI, 0.66–0.91; p > 0.05 for all paired comparisons).

**Conclusions:**

Based on current evidence, antigranulocyte scintigraphy and combined leukocyte and bone marrow scintigraphy appear to be highly specific imaging modalities in confirming periprosthetic knee infection. Bone scintigraphy was a highly sensitive imaging technique but lacks the specificity needed to differentiate among various conditions that cause painful knee prostheses. FDG-PET may not be the preferred imaging modality because it is more expensive and not more effective in confirming periprosthetic knee infection.

**Level of Evidence:**

Level III, diagnostic study.

**Electronic supplementary material:**

The online version of this article (doi:10.1007/s11999-016-5218-0) contains supplementary material, which is available to authorized users.

## Introduction

After primary TKA, as many as 2% of patients have prosthetic joint infection (PJI) develop; this risk is as great as 5% after revision surgery [3, 26] Accurate diagnosis of periprosthetic infection remains a clinical challenge, particularly in subacute or chronic infections. The evaluation of suspected PJI is characterized by a multimodality workup including microbiologic, laboratory (elevated erythrocyte sedimentation rate, C-reactive protein [CRP]), synovial marker, and histologic tests [35, 57]. Recently, promising results have been reported regarding synovial biomarkers tests, including the alpha defensin immunoassay and synovial fluid CRP tests [5, 54]. However, these test are not yet widely available and their utility has been confirmed in only a few studies [54]. In addition to these diagnostic tests, various imaging techniques including radiographs, ultrasound, CT, MRI, bone, leukocyte, bone marrow, or antigranulocyte scintigraphy, and positron emission tomography (PET) can be used in the assessment of suspected periprosthetic knee infection [10, 11, 29, 31, 57], especially in the case of a challenging diagnosis of a chronic or low-grade infection [45–48].

A delay in diagnosing and treating a periprosthetic knee infection can have a critical effect on loosening or maintaining the prosthesis and joint function. Timely identification of a periprosthetic infection is essential to allow initiation of appropriate medical and surgical therapies [49] in which various imaging modalities can contribute when other tests are inconclusive. However, inconsistent diagnostic accuracies across studies investigating periprosthetic knee infection have been published [10, 11, 22]. Consequently, the choice of the most accurate imaging technique remains controversial [11, 31]. To our knowledge, there has been no meta-analysis comparing the most commonly used imaging modalities to evaluate TKA PJI.

The aim of this systematic review and meta-analysis was to compare the diagnostic accuracy of different imaging modalities used for diagnosing periprosthetic knee infection.

## Materials and Methods

### Search Criteria and Strategy

The imaging modalities that were reviewed for the assessment of periprosthetic knee infection were radiography, ultrasound, CT, MRI, scintigraphy (including bone, antigranulocyte, leukocyte, and bone marrow scintigraphy), and PET.

**Fig. 1:**
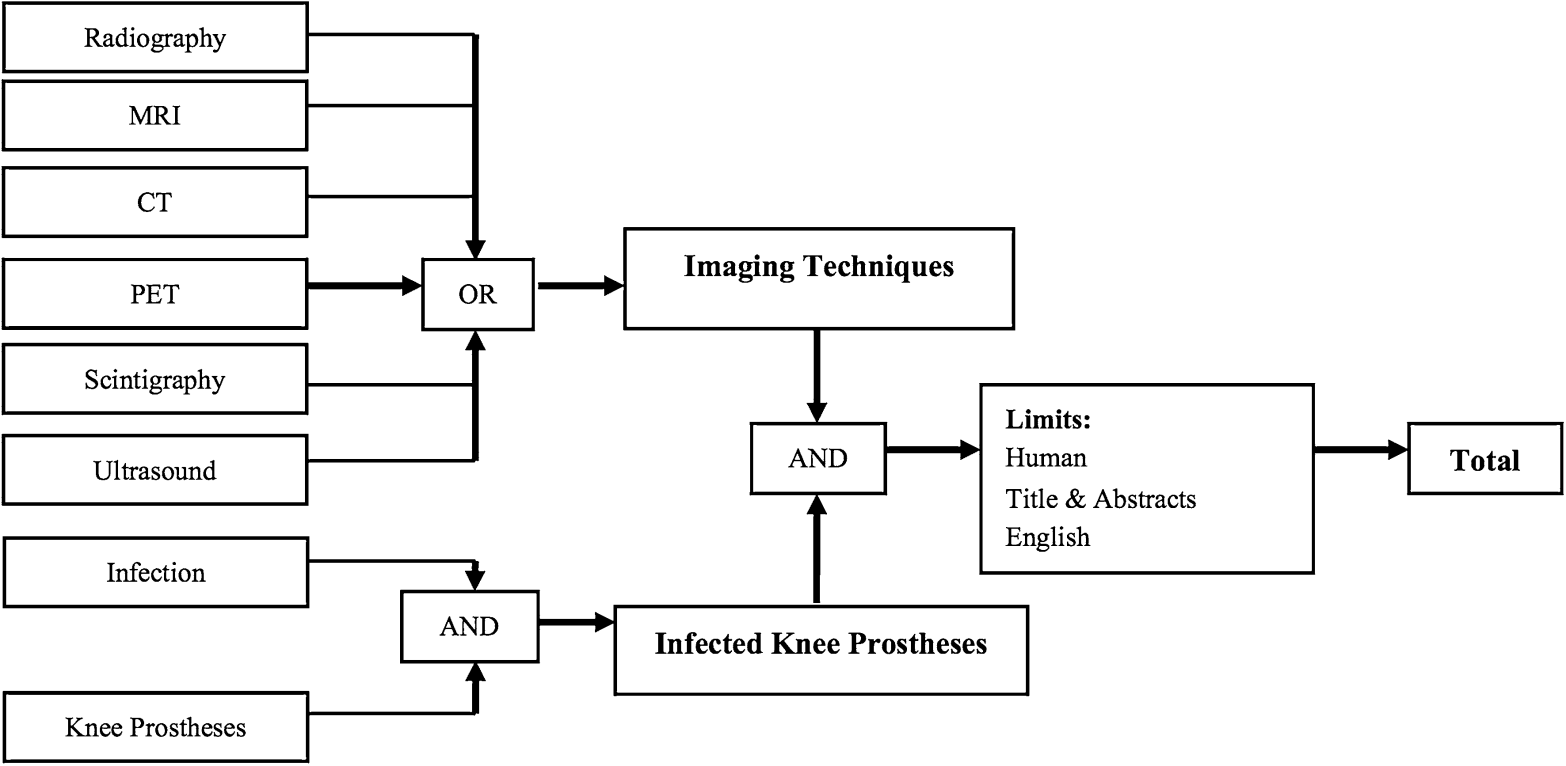
The flowchart shows the search strategy we used for this study.

In June 2015 a computer-aided search of the PubMed and Embase^®^ databases was conducted and updated in January 2016 (Appendix 1. Supplemental material is available with the online version of *CORR* ^*®*^). The search was restricted regarding primary studies that were written in English. For each database, a specific search strategy was developed (Fig. 1) with a medical informatics specialist. Reference lists of the identified studies and relevant reviews were hand-searched for supplementary eligible studies. The search was performed according to the PRISMA Statement (Appendix 2. Supplemental material is available with the online version of *CORR* ^*®*^) [24].

### Study Selection

The following inclusion criteria were used for eligible studies: (1) radiography, ultrasound, CT, MRI, scintigraphy, and PET were used to identify suspected periprosthetic knee infections; (2) a valid reference standard of positive intraoperative culture whether combined with histopathologic evidence regarding acute inflammation of the periprosthetic tissue of surgical débridement or prosthesis removal and/or the presence of a sinus tract that communicates with the prosthesis [8, 13, 29] and/or a clinical followup of at least 6 months; and (3) adequate details to reconstruct a two-by-two contingency table to determine the results of the index tests. Exclusion criteria were (1) animal studies; (2) non-English studies; (3) studies that did not differentiate between various joint replacements; and (4) case reports. Potential overlap of patient populations was assessed when more than one study was selected by the same author or institution by comparing the patient demographics. The study with the largest number of patients was selected when an overlap of patient populations between studies was observed.

The titles were screened for eligibility by one reviewer (SJV) and then processed for abstract assessment. The titles and abstracts were independently screened and assessed in an unblinded standardized manner for eligibility by two reviewers (SJV, RJAS). The final decision regarding inclusion was based on the full article. Disagreement in the evaluation of three studies was resolved with consensus by a third reviewer (OPPT). A priori, no differentiation was made for the type of knee implant, the interpretation criteria used for the index test, or the time between surgery and imaging.

### Studies Included

The search strategy identified 3708 studies from MEDLINE and 2864 studies from Embase^®^. The source population was formed by the total of 6572 studies (including duplicates). In 1933 studies, overlap was found between the retrieved studies from Embase^®^ and MEDLINE. Of the initial 6572 studies, 6433 were excluded after analyzing the information provided in the title and abstract. The full articles of the remaining 139 studies were reviewed for eligibility (Appendix 2. Supplemental material is available with the online version of *CORR* ^*®*^). No other studies were extracted from the reference list of these studies. A total of 116 studies were excluded because the study was not a clinical diagnostic study (32%), did not describe periprosthetic knee infection (12%), was not written in English (17%), did not specify the definition of positivity regarding the index test or applied an insufficient reference standard for periprosthetic knee infection (7%), did not differentiate regarding different prosthetic joint replacements (15%), did not provide data to reproduce two-by-two contingency tables (16%), or the study revealed a potential overlap of the patient population (1%). Eventually, 23 studies were included in this review.

### Description of Study Characteristics

Of the 23 studies included for meta-analysis, six used bone scintigraphy [12, 20, 27, 34, 40, 50], four used bone leukocyte scintigraphy [20, 34, 41, 50], six used leukocyte scintigraphy [18, 28, 34, 37, 38, 50], seven used leukocyte bone marrow scintigraphy [2, 7, 9, 16, 17, 21, 34], five used antigranulocyte scintigraphy [14, 15, 40, 44, 52], and five used fluorodeoxyglucose (FDG)-PET [2, 21, 23, 50, 56]. Altogether, a total of 1027 diagnostic images, 404 (39%) with and 623 (61%) without periprosthetic knee infection, were evaluated in 1502 patients with 763 knee prostheses, of which 288 (38%) were infected (Table 1). Of the studies not included for meta-analysis, two studies used ciprofloxacin scintigraphy and one used IgG scintigraphy. No studies were included that used radiographs, ultrasound, CT, MRI, or combined bone and gallium scintigraphy. The two reviewers (SJV, RJAS) independently extracted relevant data of the included studies, which included demographic, implant, and index test characteristics (Table 2). Imaging procedures, image interpretation, and the effects of time after surgery as determined by the publication data (to form subgroups when possible) were analyzed in detail, such as data regarding diagnostic performance indices (eg, sensitivity and specificity).

**Table 1:** Characteristics of the included studies

Study	Year	Design^*^	Number of patients	Patients demographics (male/female)	Age (range)	Age (mean)	Number of prostheses	Reference standard	Number of hip prostheses (total/infected)
Rand & Brown [38]	1990	Retrospective	38	16/22	26–86	65.0	38 knee	M, H, IOF	38/18
Palestro et al.[34]	1991	Retrospective	28	7/21	23–85	65.0	32 knee	M, IOF, CFU	32/9
Ooi et al. [28]	1993	NR	19	11/8	25–84	NR	6 knee	M, H, IOF, CFU	6/3
Nijhof et al. [27]	1997	NR	226	108/118	5–90	54.0	87 hip, 17 knee	M, IOF, CFU	17/3
Scher et al. [41]	2000	NR	143	NR	26–87	61.0	91 hip, 40 knee	M, H, IOF	40/14
van Acker et al. [50]	2001	Prospective	21	8/13	33–78	66.0	22 knee	M, CFU	22/6
Joseph et al. [16]	2001	Retrospective	58	18/40	27–82	60.0	36 hip, 22 knee	M, H, IOF	22/6
Larikka et al. [20]	2001	Prospective	28	4/24	47–82	75.0	30 knee	M, IOF	30/8
Zhuang et al. [56]	2001	NR	62	NR	27–81	NR	38 hip, 36 knee	M, IOF, A, CFU	36/11
Ivancevic et al. [14]	2002	Retrospective	30	13/17	30–85	Median 62.0	21 hip, 6 knee	M, H	6/2
El Espera et al.[7]	2004	NR	60	NR	NR	NR	45 hip, 28 knee	M, IOF, A	28/7
Love et al. [21]	2004	NR	59	22/37	35–89	NR	40 hip, 19 knee	M, H, IOF	19/11
Pelosi et al. [37]	2004	Retrospective	78	36/42	30–87	70.0	47 hip, 40 knee	M, IOF, CFU	40/25
von Rothenburg et al. [52]	2004	Retrospective	38	9/29	45–81	71.0	26 hip, 12 knee	M, IOF, A, L	12/4
Iyengar & Vinjamuri [15]	2005	Retrospective	38	18/20	54–89	NR	17 hip, 13 knee, 8 other	M, IOF, A, I, CFU	13/2
Stumpe et al. [44]	2006	Prospective	28	13/15	50–86	67.0/59.0	28 knee	M, CFU	28/3
Rubello et al. [40]	2008	Prospective	78	27/51	49–81	5.0	78 knee	M, CFU	78/41
Mayer-Wagner et al. [23]	2010	NR	32	13/19	45–90	NR	30 hip, 44 knee	M	16/7
Fuster et al. [9]	2011	Prospective	40	14/26	NR	66.0	21 hip, 16 knee, 3 shoulder	M, IOF, CFU	16/6
Jung et al. [17]	2012	Prospective	11	2/9	NR	72.0	11 knee	M, H, IOF, CFU	11/5
Basu et al. [2]	2014	Prospective	87	35/52	32–83	57.0	134 hip, 87 knee	M, H, IOF	87/19
Kim et al. [18]	2014	Retrospective	164	53/111	17–82	65.0	71 hip, 93 knee	M, H, IOF, CFU, A	93/63
Granados et al. [12]	2015	Prospective	120	50/70	NR	71.0	63 hip, 57 knee	M, CFU	57/8

^*^Explicit notation in study; M = microbiology; H = histology; IOF = intraoperative findings; A = aspiration; L = laboratory (erythrocyte sedimentation rate, C-reactive protein); I = imaging; CFU = clinical followup at least 6 months; NR = not recorded.

**Table 2:** Characteristics of the reference test(s) and implants

Study	Hip prostheses (primary/revision)	Cemented/uncemented	Age of knee prostheses	Imaging: minimal time after surgery	Minimal followup (months)
Rand & Brown [38]	P30, R8	C34, U2, H2	Mean 27 months (1–100 months)	> 1 month	NR
Palestro et al. [34]	NR	NR	Mean 6 years (3 weeks to 13 years)	> 3 weeks	> 6
Ooi et al. [28]	NR	NR	3–18 months	> 3 months	NR
Nijhof et al. [27]	NR	C16, U1	4.5 years (6 weeks to 21 years)	> 6 weeks	> 12
Scher et al. [41]	P40	NR	NR	Mean 71 months (median 47 months)	NR
van Acker et al. [50]	P16, R5	C12, U9	Mean 35.0 months (7 months to 9 years)	> 7 months	> 6
Joseph et al. [16]	NR	NR	NR	NR	NR
Larikka et al. [20]	NR	NR	Median 4 years 8 months (1 month to 16 years)	NR	> 12
Zhuang et al. [56]	NR	NR	3 months to 8 years	> 3 months	> 12
Ivancevic et al. [14]	NR	NR	NR	Median 13 months (2–80 months)	> 6
El Espera et al. [7]	NR	NR	Mean 5.4 years	NR	> 3
Love et al. [21]	P17, R2	C18, U1	1 week to 19 years	> 1 week	NR
Pelosi et al. [37]	NR	NR	NR	NR	> 12
von Rothenburg et al. [52]	NR	C6, U6	2 months to 10 years	> 2 months	NR
Iyengar & Vinjamuri [15]	NR	NR	NR	NR	> 12
Stumpe et al. [44]	P24, R4	C15, U13	NR	> 6 months (mean 28 [6–108 months])	> 6
Rubello et al. [40]	NR	NR	4 months to 9.5 years	> 4 months	> 12
Mayer-Wagner et al. [23]	NR	NR	NR	NR	NR
Fuster et al. [9]	NR	NR	Median 1.5 years	NR	> 12
Jung et al. [17]	P24, R4	NR	3.4 years (> 3 months	> 3 months	> 12
Basu et al. [2]	NR	NR	NR	Mean 3.7 years (FDG-PET), 6.4 (LS-BMS)	> 6
Kim et al. [18]	NR	NR	Median 3 years (2 weeks to 32 years)	NR	> 12
Granados et al. [12]	NR	NR	Average 78 months	NR	> 12

P = primary implant; R = revision; C = cemented knee prostheses; U = uncemented knee prostheses; H = hybrid hip prostheses; NR = not recorded; FDG-PET = fluorodeoxyglucose-positron emission tomography; LS-BMS = combined leukocyte and bone marrow scintigraphy.

### Methodologic Quality Assessment

The criteria list of the Quality Assessment of Diagnostic Accuracy Studies (QUADAS-2) for evaluating internal and external validity of diagnostic studies recommended by the Cochrane Screening and Diagnostic Tests Methods Group (http://methods.cochrane.org/sdt/handbook-dta-reviews) was used for grading the methodologic quality of the selected studies [53]. Evaluation was performed by two reviewers (SJV, RJAS) independently. Internal and external criteria were used for determination of the methodologic limitations, respectively, for descriptive purposes. Studies, however, were not excluded from the systematic review on the basis of quality.

**Fig. 2A–B:**
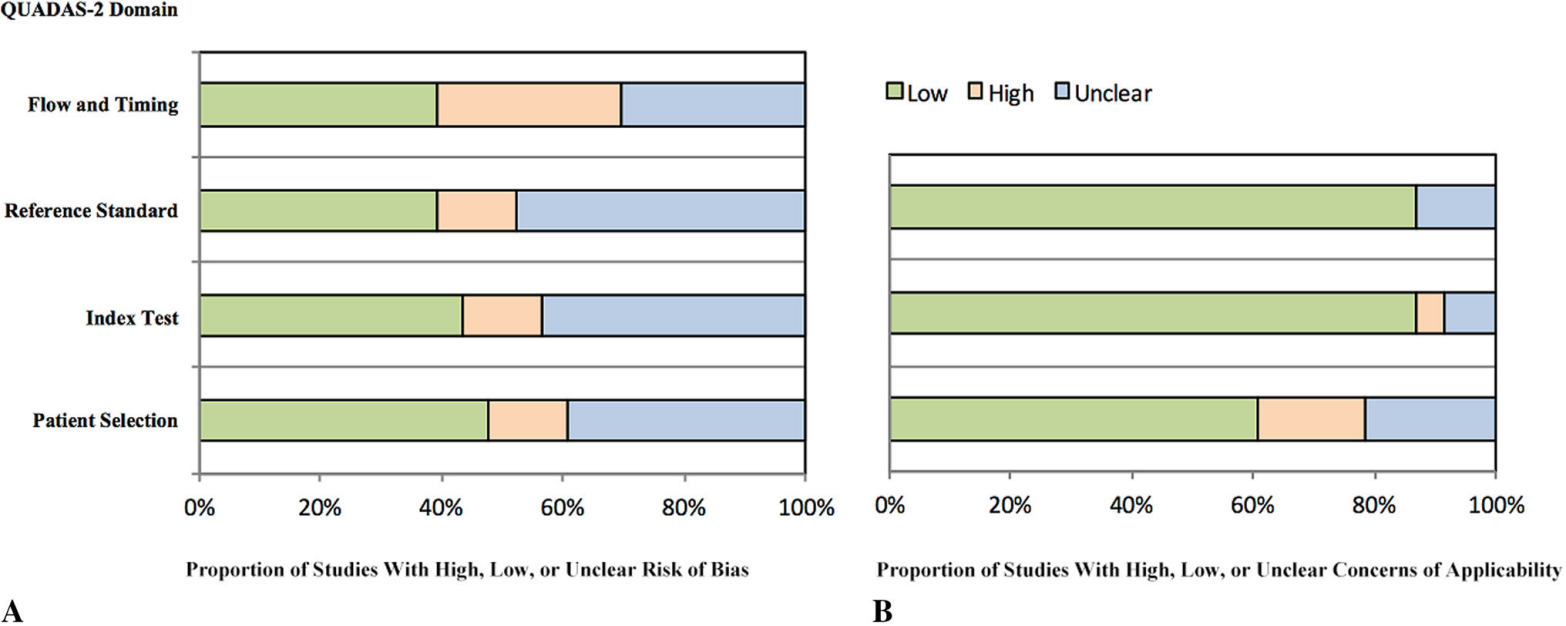
The methodologic quality of the included studies using QUADAS-2 shows the proportions of studies with high, low, or unclear **(A)** risk of bias and **(B)** concerns regarding applicability.

The external validity showed low concerns regarding applicability in more than 85% of the included studies (Fig. 2). The internal validity of the included studies showed more concerns regarding the risk of bias. Approximately 50% of the included studies did not provide sufficient information regarding patient selection, reference standard, and flow and timing.

### Quantitative Analysis (Meta-analysis)

For the diagnostic modalities, true-positive, false-positive, true-negative, and false-negative results were derived from a two-by-two contingency table. The interpretation criteria with the highest diagnostic accuracy were selected in case multiple interpretation sets for the same index test were used. When studies reported results for more than one observer, the first readers’ findings were included. The statistical heterogeneity of the diagnostic odds ratio (DOR) of each imaging index test across studies was tested using the chi-square test (Q^DOR^) for independence with *k*-1 degrees of freedom (*k* = number of studies) [6]. The Spearman rank correlation coefficient ρ value of the DOR was used in case of heterogeneity to measure the correlation between sensitivity and specificity. A ρ of 0.40 or less suggests that the variation between studies may be explained by different cutoff points, or diagnostic thresholds, on a summary receiver operating characteristic curve [6, 25]. The symmetry of funnel plots was visually interpreted to evaluate possible publication bias.

For all included studies, the test of homogeneity for the DOR indicated no statistical heterogeneity. The studies that evaluated bone scintigraphy (six studies, n = 216 knee prostheses), combined bone and leukocyte scintigraphy (four studies, n = 114 knee prostheses), leukocyte scintigraphy (six studies, n = 238 knee prostheses), combined leukocyte and bone marrow scintigraphy (seven studies, 144 knee prostheses), antigranulocyte scintigraphy (five studies, n = 136 knee prostheses), and FDG-PET (five studies, 179 knee prostheses), the Q ^DOR^ was 8.09 (5 DOF), 2.90 (3 DOF), 3.52 (5 DOF), 4.69 (6 DOF), 4.70 (4 DOF), and 6.92 (4 DOF), respectively. The funnel plots did not suggest the presence of positive-outcome bias (data not shown).

The sensitivity and specificity were pooled independently and were weighted by the inverse of the variance with use of Meta-DiSc software (Available at: http://www.hrc.es/investigacion/metadisc_en.htm) [55]. The logit-transformed sensitivity, specificity, and corresponding 95% CI of the index tests were compared with use of z-test statistics. A probability less than 0.05 was considered significant (Table 3). In the comparison of two imaging modalities, confidence intervals for two means can overlap and yet the two means can be statistically different from one another at a probability less than 0.05 [1, 19, 36]. The z-test was used to statistically analyze these differences. A secondary analysis was performed to evaluate possible influence of the methodologic quality on the sensitivity and specificity.

**Table 3:** Comparison of imaging techniques in diagnosing periprosthetic knee infection using the z-test

Imaging techniques compared		Sensitivity			Specificity		
Technique 1	Technique 2	Technique 1	Technique 2	Comparison (p value)	Technique 1	Technique 2	Comparison (p value)
BS	BS-LS	0.93	0.87	0.39	0.56	0.82	< 0.001
LS	BS-LS	0.88	0.87	0.89	0.77	0.82	0.44
LS	BS	0.88	0.93	0.34	0.77	0.56	< 0.001
LS	LS-BMS	0.88	0.80	0.24	0.77	0.93	< 0.001
LS-BMS	BS	0.80	0.93	0.08	0.93	0.56	< 0.001
LS-BMS	BS-LS	0.80	0.87	0.47	0.93	0.82	< 0.001
AGS	BS	0.90	0.93	0.60	0.95	0.56	< 0.001
AGS	BS-LS	0.90	0.87	0.70	0.95	0.82	0.01
AGS	LS	0.90	0.88	0.72	0.95	0.77	< 0.001
AGS	LS-BMS	0.90	0.80	0.21	0.95	0.93	0.47
FDG-PET	BS	0.70	0.93	0.06	0.84	0.56	< 0.001
FDG-PET	BS-LS	0.70	0.87	0.11	0.84	0.82	0.73
FDG-PET	LS	0.70	0.88	0.01	0.84	0.77	0.21
FDG-PET	LS-BMS	0.70	0.80	0.30	0.84	0.93	< 0.001
FDG-PET	AGS	0.70	0.90	0.02	0.84	0.95	0.02

BS = bone scintigraphy; LS = leukocyte scintigraphy; BMS = bone marrow scintigraphy; AGS = antigranulocyte scintigraphy; FDG-PET = fluorodeoxyglucose-positron emission tomography.

## Results

Bone scintigraphy was less specific (Table 3) than all other modalities tested (56%; 95% CI, 0.47–0.64; p < 0.001), and leukocyte scintigraphy (77%; 95% CI, 0.69–0.85) was less specific than antigranulocyte scintigraphy (95%; 95% CI, 0.88–0.98; p < 0.001) or combined leukocyte and bone marrow scintigraphy (93%; 95% CI, 0.86–0.97; p < 0.001). FDG-PET (84%; 95% CI, 0.76–0.90) was more specific than bone scintigraphy (56%; 95% CI, 0.47–0.64; p < 0.001), and less specific than antigranulocyte scintigraphy (95%; 95% CI, 0.88–0.98; p = 0.02) and combined leukocyte and bone marrow scintigraphy (93%; 95% CI, 0.86–0.97; p < 0.001).

Leukocyte scintigraphy (88%; 95% CI, 0.81–0.93; p = 0.01) and antigranulocyte scintigraphy (90%; 95% CI, 0.78–0.96; p = 0.02) were more sensitive than FGD-PET (70%; 95% CI, 0.56–0.81). However, because of broad overlapping of confidence intervals, no differences in sensitivity were observed among the other modalities, including combined bone scintigraphy (93%; 95% CI, 0.85–0.98) or combined leukocyte and bone marrow scintigraphy (80%; 95% CI, 0.66–0.91; p > 0.05 for all paired comparisons (Table 3).

The secondary analysis, when high risk of bias studies were excluded, showed a higher sensitivity for FDG-PET (93%; 95% CI, 0.80–0.98) that was not different than leukocyte scintigraphy (86%; 95% CI, 0.76–0.93; p = 0.39) and antigranulocyte scintigraphy (91%; 95% CI, 0.78–0.98; 0.18). Combined leukocyte and bone marrow scintigraphy was highly specific (92%; 95% CI, 0.84–0.97) and more specific than bone scintigraphy (55%; 95% CI, 0.45–0.64; p ≤ 0.001) and leukocyte scintigraphy (71%; 95% CI, 0.56–0.84; p = 0.01). However, antigranulocyte scintigraphy (98%; 95% CI, 0.92–0.99) was more specific than all other compared imaging modalities; p < 0.05 for all paired comparisons.

## Discussion

**Fig. 3A–B:**
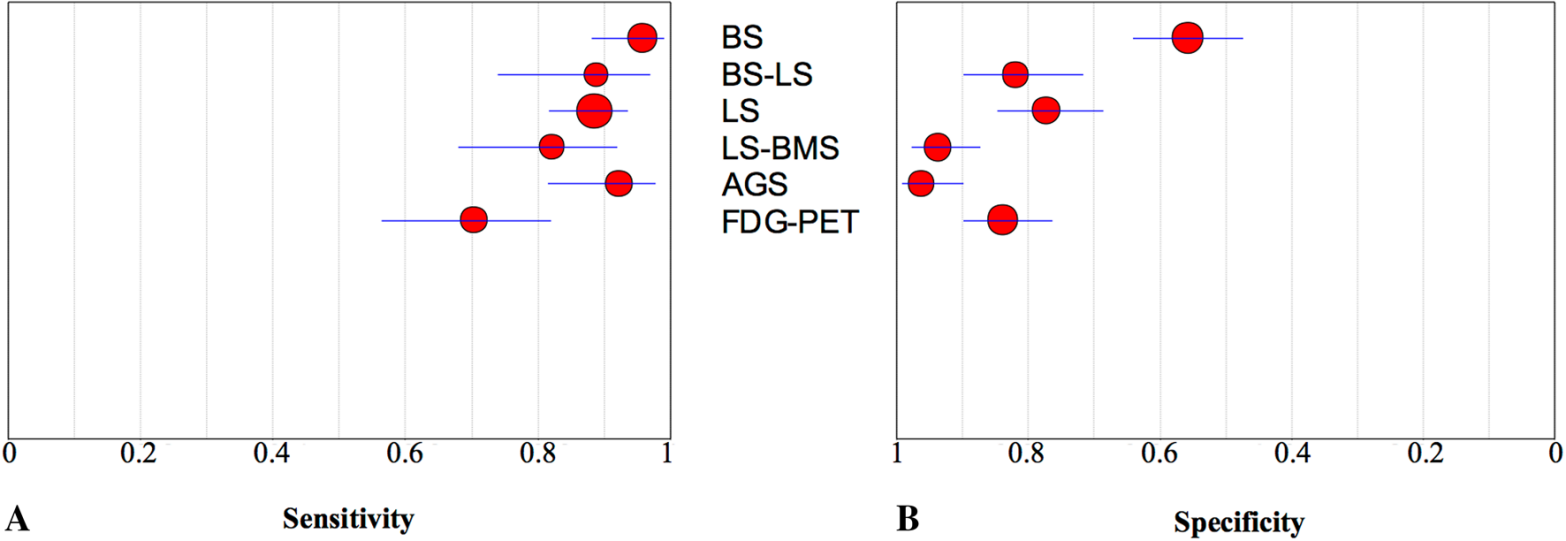
The graphs show the pooled estimates and corresponding 95% CIs for **(A)** sensitivity and **(B)** specificity for all index tests. The size of the circles is proportionate to the number of patients investigated by each technique. BS = bone scintigraphy; BS/LS = bone and leukocyte scintigraphy; LS = leukocyte scintigraphy; LS/BMS = leukocyte and bone marrow scintigraphy; AGS = antigranulocyte scintigraphy; FDG-PET = fluorodeoxyglucose positron emission tomography.

In the assessment of suspected periprosthetic knee infection, various diagnostic tests including blood tests, synovial fluid microbiologic analyses, and synovial fluid marker tests (such as alpha defensin and synovial fluid CRP), can be used. However, accurate diagnosis of periprosthetic knee infection remains challenging, especially in chronic or low-grade infections, and inconsistent diagnostic accuracies with various tests across studies have been published [10, 11, 22]. Because of that, imaging tests remain important, but studies do not agree on which imaging technique is the most accurate [11, 31]. Our meta-analysis revealed that in diagnosing periprosthetic knee infection, antigranulocyte scintigraphy and combined leukocyte and bone marrow scintigraphy were highly specific imaging techniques (Fig. 3).

Although the included studies showed statistical homogeneity of data, the reliability of the pooled estimates depends on the methodologic quality of the included studies. There are several limitations of this meta-analysis to consider. Collecting large sample sizes of patients with suspected periprosthetic knee infection is difficult; the total number of infected TKAs included in this meta-analysis was only 288. Subsequently, several studies showed wide confidence intervals, because of small numbers of patients who were evaluated using each diagnostic modality. This means that there may have been differences in sensitivity or specificity between certain modalities that we did not detect. Future comparative studies might help resolve this issue. Studies were not excluded on the basis of methodologic quality. Our secondary analysis, with exclusion of studies that showed a high risk of bias, suggested that FDG-PET might be more sensitive than the primary analysis showed; indeed, it may be comparably sensitive to leukocyte scintigraphy and antigranulocyte scintigraphy. The methodologic quality of the included studies did not substantially influence the sensitivity and specificity of other imaging modalities (data not shown). However, there were important concerns regarding the flow and timing of the included studies. Most of the studies often insufficiently described important variables, including types of implants, use of antibiotics, imaging time after surgery, improvement of imaging techniques, and inter- and intraobserver reliability variance. Consequently, analyses of the effect of these variables on the accuracy of imaging was not possible, but could influence the diagnostic performance of the imaging modalities we studied. In addition, the long period evaluated here (1990 to 2015) saw the introduction of numerous new diagnostic tests (such as alpha defensin and synovial fluid CRP) and new diagnostic standards [4], which might have changed the apparent performance of the imaging modalities we studied and how they might be used in practice. The differentiation between acute or chronic infection influences the decision to evaluate a suspected infection with additional imaging, and should be investigated in additional studies.

Another important limitation of the included studies is the lack of uniform criteria for diagnosis of a periprosthetic infection. We could not restrict inclusion to studies using the Musculoskeletal Infection Society criteria [35] because many of the included studies were performed before the development of these criteria. Although a valid reference standard with microbiologic confirmation was a stringent inclusion criterion in this meta-analysis, there is a risk of false-positive diagnosis of infection, which potentially could decrease specificity. When a diagnosis of no infection was considered, clinical followup sometimes was used to monitor the final diagnosis. Only studies with a clinical followup of at least 6 months were included. For obvious reasons, surgery with microbiologic evaluation could not be performed in all patients (patients believed to be without infection did not always undergo surgery). However, this could result in more false-negative results and potentially decrease the reported specificity when an infection is found after the final diagnosis, especially in the case of a low-grade infection.

Our meta-analysis defined test performance for the various imaging modalities when used in isolation. However, multiple diagnostic tests including aspiration results and laboratory tests can contribute in diagnosing periprosthetic infection, which could influence the diagnostic performance of the evaluated imaging techniques, and generally should improve their performance. During the years, important developments have been described in the diagnosis of periprosthetic infection, including the introduction of alpha defensin and synovial fluid tests [5, 54]. When the diagnostic evaluation using synovial fluid markers clearly indicates infection, there is little or no need for additional nuclear imaging tests. However, if those tests cannot be obtained or are inconclusive, nuclear imaging can be used in concert with other elements of diagnostic evaluation, including microbiologic analysis and blood testing, to arrive at a more-precise diagnosis than is possible with imaging or laboratory testing alone. Nuclear imaging seldom is used in isolation, and probably should not be used that way [57].

**Fig. 4A–B:**
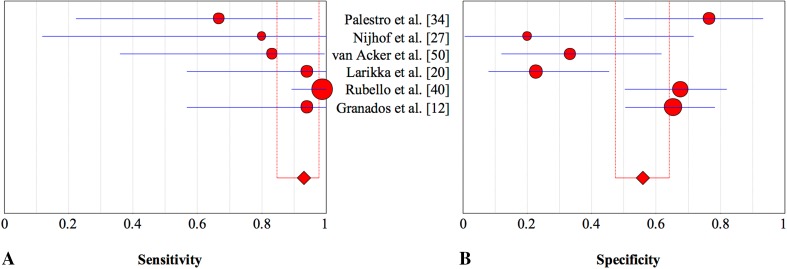
The pooled **(A)** sensitivity and **(B)** specificity of bone scintigraphy in the assessment of periprosthetic knee infection with 95% CIs are presented.

Using bone scintigraphy during the first years after implantation, postoperative tracer (Table 4) uptake can be caused by various factors and therefore lacks the specificity needed to differentiate between aseptic and septic loosening [10, 32]. Our results (Table 5) confirmed the reputation of high sensitivity and low specificity of this technique [30, 31, 42, 43]. Unfortunately, subgroup analysis of imaging time after implantation could not be performed owing to insufficient data. In clinical practice, imaging often is used to rule out an infection. Bone scintigraphy is widely available and a sensitive tool for evaluation of painful knee prostheses (Fig. 4). However, when confirmation of infection is needed, a positive bone scintigraphy outcome usually leads to a second, more-specific, investigation.

**Table 4:** Study characteristics of bone scintigraphy for detection of periprosthetic knee infection

Study	Tracer	Doses	Criteria for infection
Palestro et al. [34]	99mTc-MDP	740 MBq	When hyperperfusion and hyperemia around at least one component of the prosthesis were present on dynamic and blood pool images and periprosthetic activity of at least Grade 2 around the same component was present on delayed images
Nijhof et al. [27]	99mTc-MDP	600 MBq	When there was increased activity in at least two phases (blood pool and late phase) in the area of interest
van Acker et al. [50]	99mTc-MDP	740 MBq	Any periprosthetic focal uptake in the delayed phase
Larikka et al. [20]	99mTc-HDP	550 MBq	Uptake in the arterial/flow phase
Rubello et al. [40]	99mTc-MDP	NR	Uptake in the arterial/flow phase
Granados et al. [12]	99mTc-HDP	925 MBq	Uptake in the arterial/flow phase

Diagnostic odds ratio 8.956; heterogeneity chi-square = 8,09 (df = 5) p = 0.151; Inconsistency (I^2^) = 38,2%; 99 mTc = 99m-technetium; MDP = methylenediphosphonate; HDP = hydroxymethylenediphosphonate.

**Table 5:** Diagnostic accuracy of bone scintigraphy for detection of periprosthetic knee infection

Study	Year	Disease		Sensitivity	95% CI	Specificity	95% CI
		+	−				
Palestro et al. [34]	1991	6	17	0.67	0.22–0.96	0.76	0.50–0.93
Nijhof et al. [27]	1997	2	5	1.00	0.12–1.00	0.20	0.00–0.72
van Acker et al. [50]	2001	6	15	0.83	0.36–1.00	0.33	0.12–0.62
Larikka et al. [20]	2001	8	22	1.00	0.57–1.00	0.23	0.08–0.54
Rubello et al. [40]	2008	41	37	1.00	0.89–1.00	0.68	0.50–0.82
Granados et al. [12]	2015	8	49	1.00	0.94–1.00	0.65	0.50–0.78
Total		71	145				
Pooled estimate				0.93	0.85–0.98	0.56	0.47–0.64

**Fig. 5A–B:**
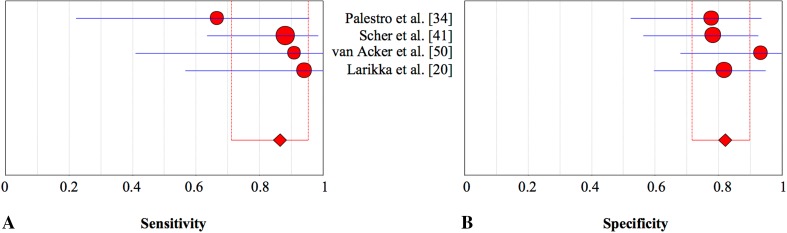
The pooled **(A)** sensitivity and **(B)** specificity of leukocyte scintigraphy in the assessment of periprosthetic knee infection with 95% CIs are shown.

**Fig. 6A–B:**
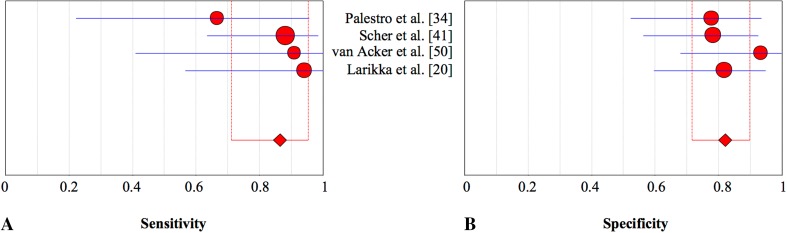
The pooled **(A)** sensitivity and **(B)** specificity of combined bone and leukocyte scintigraphy in the assessment of periprosthetic knee infection with 95% CIs are presented.

Leukocyte scintigraphy is assumed to be a more specific-imaging modality and has a long history of use in detection of infections [11, 51]. However, our meta-analysis showed that this technique alone may not be the preferred modality for confirming periprosthetic knee infection, given that it has only moderate specificity (77%) (Table 6). We found that leukocyte scans are very sensitive (88%) (Fig. 5). However, in contrast to bone scintigraphy, leukocyte scintigraphy is a time-consuming procedure with higher costs and therefore may not be the preferred imaging technique to rule out periprosthetic knee infection. The explanation for the moderate specificity may be that labeled leukocytes (Table 7) not only accumulate in infections, but also physiologically in the bone marrow [33]. To reduce the consequent number of false-positive results, leukocyte scintigraphy can be combined with bone marrow scintigraphy (Table 8), which has been proposed as the preferred imaging modality for diagnosing prosthetic joint infections [10, 11, 22, 32]. The current results for knee prostheses confirmed an increased specificity of 93% versus 77% when combining leukocyte with bone marrow scintigraphy (Table 9). Another assessed option to improve specificity (Table 10) was combining leukocyte with bone scintigraphy (Table 11). As expected, specificity did not improve (Fig. 6) [10]. More recently, antigranulocyte scintigraphy was introduced as a less time-consuming alternative for leukocyte scintigraphy with the advantage of in vivo labeling of leukocytes with considerable potential in the detection of infection (Table 12) [10, 11]. We found antigranulocyte scintigraphy (Table 13) to be more specific than leukocyte scintigraphy and FGD-PET (Table 14). However, its role in the assessment of periprosthetic infection is not yet fully established [10]. An important drawback in clinical practice is that neither antigranulocyte scintigraphy nor leukocyte scintigraphy are widely available and used in clinical practice [10].

**Table 6:** Diagnostic accuracy of leukocyte scintigraphy for detection of periprosthetic knee infection

Study	Year	Disease		Sensitivity	95% CI	Specificity	95% CI
		+	−				
Rand & Brown [38]	1990	18	20	0.83	0.59–0.96	0.85	0.62–0.97
Palestro et al. [34]	1991	9	32	0.89	0.52–1.00	0.75	0.57–0.89
Ooi et al. [28]	1993	3	3	1.00	0.23–1.00	0.67	0.09–0.99
van Acker et al. [50]	2001	5	15	1.00	0.41–1.00	0.53	0.27–0.79
Pelosi et al. [37]	2004	25	15	0.96	0.80–1.00	0.93	0.68–1.00
Kim et al. [18]	2014	63	30	0.86	0.75–0.93	0.80	0.61–0.92
Total		123	115				
Pooled estimate				0.88	0.81–0.93	0.77	0.69–0.85

**Table 7:** Study characteristics of leukocyte scintigraphy for detection of periprosthetic knee infection

Study	Tracer	Doses^*^	Criteria for infection
Rand & Brown [38]	111 In-Oxine	NR	A focus of moderate to marked increased activity
Palestro et al. [34]	111 In-Oxine	18.5 MBq	Periprosthetic activity more intense than that in corresponding contralateral region
Ooi et al. [28]	111 In-Oxine	7.5–15 MBq	When there was a focal accumulation at the area of interest
van Acker et al. [50]	99Tc-HMPAO	185 MBq	Any periprosthetic focal uptake
Pelosi et al. [37]	99Tc-HMPAO	430–600 MBq	SQ: K_late_ > K_early_ by at least 10%
Kim et al. [18]	99Tc-HMPAO	740–1100 MBq	Increased uptake in the periprosthetic area or if the foci in nearby soft tissue had greater activity than the background soft tissue activity

^*^Mean doses; SQ = semiquantitative; diagnostic odds ratio 28,143; heterogeneity chi-square = 3.52 (df = 5); p = 0.620; inconsistency (I^2^) = 0.0%; K = suspected region of infection/reference region (bone marrow); HMPAO = hexamethylpropyleneamine oxime; NR = not reported.

**Table 8:** Study characteristics of combined leukocyte and bone marrow scintigraphy

Study	Tracer	Doses	Criteria for infection
Palestro et al. [34]	111In-Oxine/99mTc-SC	18.5 MBq/370 MBq	If activity was observed on labeled leukocyte images without corresponding activity on the sulfur colloid images (incongruent)
Joseph et al. [16]	111In-Oxine/99mTc-SC	500 uCi /10 uCi	Pattern of activity on the indium image that was not matched on the colloid images
El Espera et al. [7]	111In-Oxine/99mTc-SC	30 MBq/185 MBq	When increased activity was observed on the leukocyte image at the ROI, without corresponding uptake on the bone marrow images (incongruent patterns)
Love et al. [21]	111In-Oxine/99mTc-SC	18.5 MBq/370 MBq	Periprosthetic activity on the indium image without corresponding activity on the marrow scan, regardless of intensity or location
Fuster et al. [9]	99Tc-HMPAO/99mTc-SC	NR	Global imaging analysis; BMS scan inconsistent with LS
Jung et al. [17]	99mTc-HMPAO /99mTc-phytate	555–740 MBq/185 MBq	Concordant if the distribution of the two radiotracers was spatially identical, and discordant if activity was observed in the LS without corresponding activity in the BMS (discordant was positive for detection of periprosthetic knee infection)
Basu et al. [2]	111In-Oxine/99mTc-SC	500 uCi/555 MBq	When activity in the periprosthetic region on the leukocyte image was observed, without corresponding activity on the bone marrow images

Diagnostic odds ratio 41,063: heterogeneity chi-square = 4.69 (df = 6), p = 0.584, inconsistency (I^2^) = 0.0%; 99mTc-SC = 99mTc-sulfur colloid; HMPAO = hexamethylpropyleneamine oxime; ROI = region of interest; LS = leukocyte scintigraphy; BMS = bone marrow scintigraphy; NR = not reported.

**Table 9:** Diagnostic accuracy of combined leukocyte and bone marrow scintigraphy for detection of periprosthetic knee infection

Study	Year	Disease		Sensitivity	95% CI	Specificity	95% CI
		+	−				
Palestro et al. [34]	1991	7	12	0.86	0.42–0.97	1.00	0.68-1.00
Joseph et al. [16]	2001	6	16	0.67	0.22–0.96	1.00	0.75–1.00
El Espera et al. [7]	2004	7	21	0.71	0.29–0.96	0.95	0.76–1.00
Love et al. [21]	2004	11	8	1.00	0.66–1.00	1.00	0.57–1.00
Fuster et al. [9]	2011	6	10	0.83	0.36–1.00	0.90	0.56–1.00
Jung et al. [17]	2012	5	6	1.00	0.41–1.00	0.83	0.36–1.00
Basu et al. [2]	2014	26	3	0.33	0.01–0.91	0.88	0.70–0.98
Total		68	76				
Pooled estimate				0.80	0.66–0.91	0.93	0.86–0.97

**Table 10:** Diagnostic accuracy of combined bone and leukocyte scintigraphy for detection of periprosthetic knee infection

Study	Year	Disease		Sensitivity	95% CI	Specificity	95% CI
		+	−				
Palestro et al. [34]	1991	6	18	0.67	0.23–0.96	0.78	0.53–0.94
Scher et al. [41]	2000	17	23	0.88	0.64–0.99	0.78	0.56–0.93
van Acker et al. [50]	2001	5	15	1.00	0.41–1.00	0.93	0.68–1.00
Larikka et al. [20]	2001	8	22	1.00	0.56–1.00	0.82	0.60–0.95
Total		36	78				
Pooled estimate				0.87	0.71–0.96	0.82	0.72–0.90

**Table 11:** Study characteristics of combined bone and leukocyte scintigraphy for detection of periprosthetic knee infection

Study	Tracer	Doses	Criteria for infection
Palestro et al. [34]	99mTc-MDP/111 In-Oxine	740 MBq/18.5 MBq	If the distribution of periprosthetic activity on leukocyte images was spatially similar to the distribution of activity on bone images but relatively more intense or if the spatial distribution of the two traces was different (incongruent)
Scher et al. [41]	99mTc-HDP/111 In-Oxine	925 MBq/14.8–18.5 MBq	When indium scan showed hyperactivity in a different distribution (incongruency) or a relatively greater intensity than the activity on the Tc99 scan
van Acker et al. [50]	99mTc-MDP/9mTc-HMPAO	740 MBq/185 MBq	Lesions on white blood cell scan that also were found on the third phase of the bone scan
Larikka et al. [20]	99mTc-HDP/99mTc-HMPAO	550 MBq/370 MBq	When periprosthetic leukocyte uptake intensity was higher than that of the bone metabolic image in at least one zone, or if uptake was incongruent

Diagnostic odds ratio 23,869: heterogeneity chi-square = 2.90 (df = 3); p = 0.408; inconsistency (I^2^) = 0.0%; Tc99 m = 99 m-technetium; MDP = methylenediphosphonate; HDP = hydroxymethylenediphosphonate; 99Tc-HMPAO = hexamethylpropyleneamine oxime.

**Table 12:** Study characteristics of antigranulocyte scintigraphy for detection of periprosthetic knee infection

Study	Antibody type	99mTc doses	Criteria for infection
Ivancevic et al. [14]	Sulesomab^′2^	< 1.1 GBq	Uptake higher than that in the bone marrow of the contralateral iliac crest
von Rothenburg et al. [52]	Sulesomab^2^	15–25 mCi	When there was abnormal uptake greater than could be expected from a blood pool effect (Q)
Iyengar & Vinjamuri [15]	Sulesomab^2^	650 MBq	Uptake in the ROI was greater than the uptake in the surrounding normal tissue or the contralateral side
Stumpe et al. [44]	Besilesomab^1^	NR	If the intensity of accumulation around the BPI exceeded physiologic bone marrow uptake or if the intensity of radionuclide uptake increased from 4–24 hours
Rubello et al. [40]	Sulesomab^2^	740 MBq	Dual interpretation (early and delayed): if on the delayed leukoscan imaging, the uptake increased by one step or more in comparison to the baseline (early leukoscan)

Diagnostic odds ratio 84,598, heterogeneity chi-square = 4.70 (df = 4), p = 0.320, inconsistency (I^2^) = 14.9%; SQ = semiquantitative; Q = qualitative; ROI = region of interest; BPI = bone-prosthesis interface; ^1^99mTc-anti-NCA 95; ^2^99mTc-anti-NCA90.

**Table 13:** Diagnostic accuracy of antigranulocyte scintigraphy for detection of periprosthetic knee infection

Study	Year	Disease		Sensitivity	95% CI	Specificity	95% CI
		+	−				
Ivancevic et al. [14]	2002	2	3	1.00	0.12–1.00	0.67	0.10–0.99
von Rothenburg et al. [52]	2004	4	8	1.00	0.33–1.00	1.00	0.57–1.00
Iyengar & Vinjamuri [15]	2005	2	11	1.00	0.12–1.00	0.82	0.48–0.98
Stumpe et al. [44]	2006	3	25	0.67	0.10–0.99	1.00	0.83–1.00
Rubello et al. [40]	2008	41	37	0.93	0.80–0.99	1.00	0.88–1.00
Total		52	84				
Pooled estimate				0.90	0.78–0.96	0.95	0.88–0.98

**Table 14:** Diagnostic accuracy of fluorodeoxyglucose-positron emission tomography for detection of periprosthetic hip infection

Study	Year	Disease		Sensitivity	95% CI	Specificity	95% CI
		+	−				
van Acker et al. [50]	2001	6	15	1.00	0.47–1.00	0.73	0.45–0.92
Zhuang et al. [56]	2001	11	25	0.91	0.59–1.00	0.72	0.51–0.88
Love et al. [21]	2004	11	8	0.27	0.06–0.61	1.00	0.57–1.00
Mayer-Wagner et al. [23]	2010	7	9	0.14	0.00–0.58	0.89	0.52–1.00
Basu et al. [2]	2014	19	68	0.95	0.74–1.00	0.88	0.78–0.95
Total		54	125				
Pooled estimate				0.70	0.56–0.81	0.84	0.76–0.90

**Fig. 7A–B:**
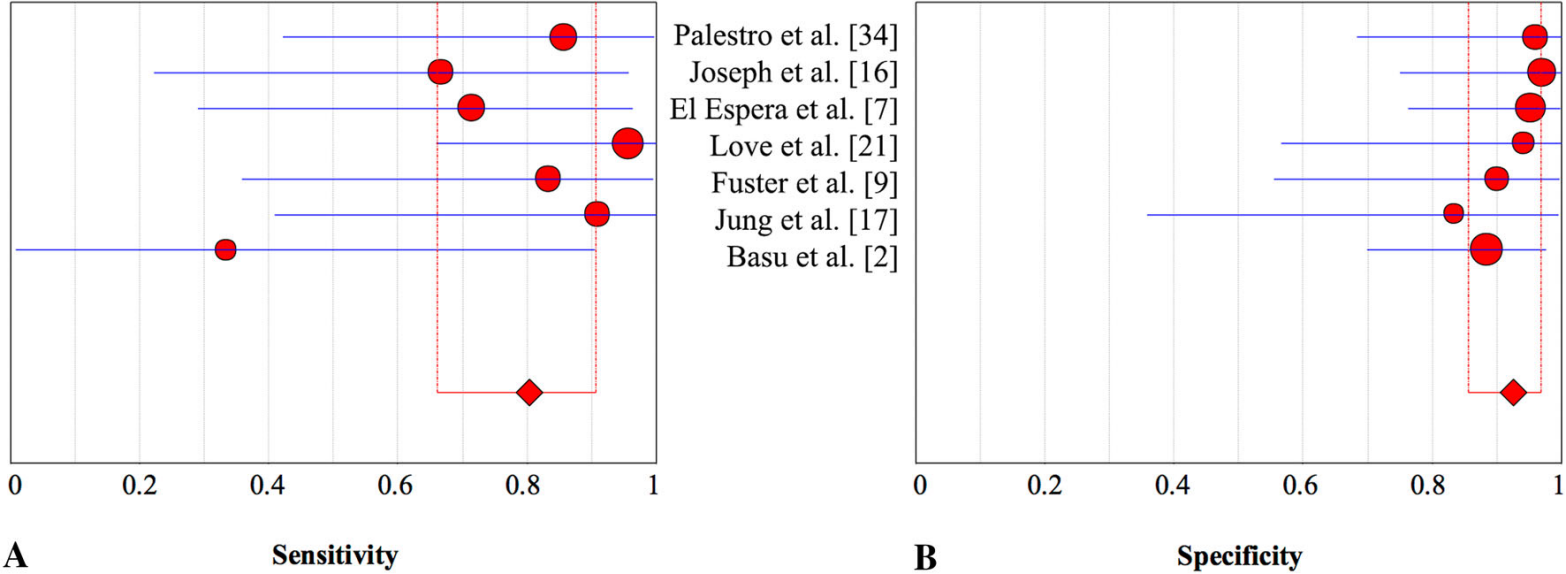
The pooled **(A)** sensitivity and **(B)** specificity of combined leukocyte and bone marrow scintigraphy in the assessment of periprosthetic knee infection with 95% CIs are presented.

**Fig. 8A–B:**
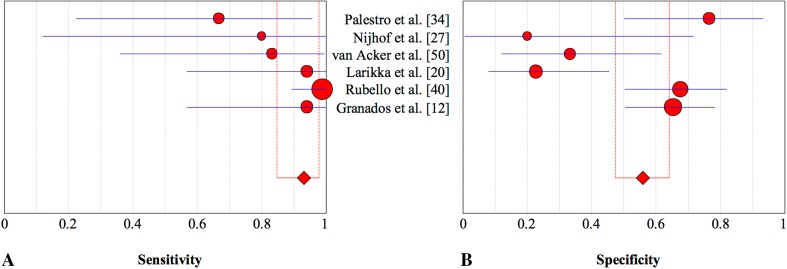
The pooled **(A)** sensitivity and **(B)** specificity of FDG-PET in the assessment of periprosthetic knee infection with 95% CIs are shown. FDG-PET = fluorodeoxyglucose-positron emission tomography.

**Fig. 9A–B:**
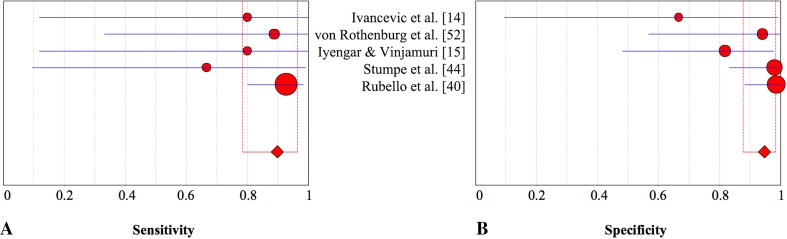
The pooled **(A)** sensitivity and **(B)** specificity of antigranulocyte scintigraphy in the assessment of periprosthetic knee infection with 95% CIs are shown.

FDG-PET is increasingly used and has proposed potential in the diagnosis of PJI, especially regarding hip arthroplasty [10, 39, 51, 58]. Although this technique offers advantages such as time efficiency, increased resolution, and the use of low-dose CT, our results revealed that this technique was less specific in diagnosing periprosthetic knee infection than combined leukocyte and bone marrow scintigraphy and antigranulocyte scintigraphy (Fig. 7). Some investigations concluded that uptake patterns rather than intensity in the bone-prosthesis interface are specific in diagnosing periprosthetic infection (Table 15) [2, 56]. In particular, the sensitivity of 70% is only moderate (Fig. 8) and was lower than the sensitivity of leukocyte or antigranulocyte scintigraphy (Fig. 9). However, our secondary analysis revealed that FDG-PET was highly sensitive (93%) when low-quality studies were excluded [21, 23], which is not less sensitive than the other imaging techniques evaluated. This should be considered further in well-designed studies. The specificity was not higher than that of combined leukocyte and bone marrow scintigraphy and antigranulocyte scintigraphy. An important drawback of FDG-PET is the high cost compared with other imaging modalities. Therefore, FDG-PET may not be the preferred imaging modality in the evaluation of a suspected infected knee prosthesis.

**Table 15:** Study characteristics of fluorodeoxyglucose positron emission tomography for detection of periprosthetic hip infection

Study	Tracer	Doses	Criteria for infection
van Acker et al. [50]	18F-FDG	3.7*Kg/8 MBq	Focal FDG uptake at the BPI
Zhuang et al. [56]	18F-FDG	4.22–4.56 MBq/kg	When an area of increased uptake was detected in the BPI, compared with adjacent soft tissue
Love et al. [21]	18F-FDG	150–220 MBq	Semiquantitative analysis of bone-prosthesis-interface (target-background ratio)
Mayer-Wagner et al. [23]	18F-FDG	180 MBq	Increased uptake at the distal BPI of the femoral shield and/or of the stem of the tibial prosthesis
Basu et al. [2]	18F-FDG	0.14 mCi/kg	Only uptake in the BPI

Diagnostic odds ratio 19,083: heterogeneity chi-square = 6.92 (df = 4), p = 0.140, inconsistency (I^2^) = 42.2%; 18F-FDG = fluorodeoxyglucose (18F-FDG); BPI = bone-prosthesis interface.

This meta-analysis revealed that, based on current evidence, antigranulocyte scintigraphy and combined leukocyte and bone marrow scintigraphy were highly specific in confirming periprosthetic knee infection. However, the time-consuming procedures and limited availability are important drawbacks of these techniques. Bone scintigraphy was highly sensitive but lacks the specificity in differentiating between various conditions of painful knee prostheses. FDG-PET may not be the preferred imaging modality because it is more expensive and not more effective in confirming infected knee prostheses. In practice, other tests should be used in concert with the evaluated imaging modalities to arrive at more-sensitive and specific diagnostic decisions than are possible with imaging or laboratory testing alone. Future, larger prospective studies should assess the utility of imaging in the diagnostic algorithm of a suspected periprosthetic knee infection, providing more data to evaluate important variables, including the differentiation between acute and chronic infections.

## Electronic supplementary material

Below is the link to the electronic supplementary material.

Supplementary material 1 (DOC 35 kb)

Supplementary material 2 (DOC 72 kb)
