# Supplementary material for: What is the Accuracy of Nuclear Imaging in the Assessment of Periprosthetic Knee Infection? A Meta-analysis
Source: Clin Orthop Relat Res. 2017 Jan 3;475(5):1395–410. doi: 10.1007/s11999-016-5218-0 (PMC5384926; doi:10.1007/s11999-016-5218-0)
Supplement: Supplementary file 1 — Supplementary material 1 (DOC 35 kb) [file 11999_2016_5218_MOESM1_ESM.doc]

Appendix 1. Search string used for identifying studies on diagnostic imaging in diagnosing periprosthetic knee infection.

**MEDLINE**

((((((((((((((((("Infection"[Mesh]) OR "Biofilms"[Mesh]) OR "Bacterial Adhesion"[Mesh]) OR "Adhesins, Bacterial"[Mesh]) OR "Bacterial Infections"[Mesh])) OR infect*[tiab]) OR sepsi*[tiab]) OR septi*[tiab]) OR bacterie*[tiab]) OR bacteria*[tiab]) OR biofilm*[tiab]) OR (bacterem*[tiab] OR bacteraem*[tiab]))) OR inflammat*[tiab])) AND (((((("Knee"[Mesh] OR "Knee Joint"[Mesh]) OR "Femur"[Mesh]) OR "Tibia"[Mesh]) OR (knee[tiab]) OR (femur*[tiab]) OR (femoral[tiab]) OR (tibia*[tiab]))))) AND ((((((((((((((("Prostheses and Implants"[Mesh:noexp]) OR "Joint Prosthesis"[Mesh:noexp]) OR "Knee Prosthesis"[Mesh]) OR "Metal-on-Metal Joint Prostheses"[Mesh]) OR "Prosthesis Implantation"[Mesh:noexp]) OR "Arthroplasty, Replacement"[Mesh:noexp])) OR prosthe*[tiab]) OR implant*[tiab]) OR arthroplast*[tiab]) OR replac*[tiab])) OR metal-on-metal[tiab]))) AND ((((("Magnetic Resonance Imaging"[Mesh] OR ("magnetic resonance"[tiab] AND (image[tiab] OR images[tiab] OR imaging[tiab])) OR zeugmatograph*[tiab] OR MRI*[tiab] OR NMR*[tiab] OR Magnetization*[tiab] OR Magnetisation*[tiab] OR fMRI*[tiab] OR MR imag*[tiab])) OR (Magnetic Resonance Spectroscopy[Mesh] AND (chemical shift[tiab] AND imag*[tiab])))) OR ((((("Tomography, X-Ray Computed"[Mesh]) OR (compute*[tiab] AND tomograph*[tiab])) OR (CT[tiab] OR CTs[tiab] OR CT's[tiab])) OR electron beam[tiab]) OR Tomodensitometr*[tiab])) OR (((((((((((((("Radiography"[Mesh]) OR "radiography"[Subheading])) OR radiograph*[tiab]) OR x-ray*[tiab]) OR x-radiograph*[tiab]) OR xray*[tiab]) OR Zonograph*[tiab]) OR fluoroscop*[tiab]) OR arthrograph*[tiab]) OR roentgen*[tiab]) OR rontgen*[tiab])) OR image subtraction*[tiab]) OR ((((((((((("Radionuclide Imaging"[Mesh]) OR "radionuclide imaging"[Subheading]) OR "Radiopharmaceuticals"[Pharmacological Action])) OR (scintigraph*[tiab] OR scintiphotograph*[tiab])) OR (radionuclid*[tiab] OR radioisotop*[tiab])) OR Gamma Camera Imag*[tiab]) OR (indium[tiab] OR in-111[tiab] OR 111in*[tiab])) OR (99mtc[tiab] OR 99m[tiab]) OR scintiscan*[tiab]))) OR ((((((((((("Positron-Emission Tomography"[Mesh]) OR "Tomography, Emission-Computed"[Mesh])) OR "Tomography"[Mesh:noexp])) OR PET[tiab]) OR tomograph*[tiab]) OR ((("Fluorodeoxyglucose F18"[Mesh]) OR "Deoxyglucose"[Mesh]))) OR (deoxyglucose[tiab] OR deoxy-glucose[tiab] OR 2deoxyglucose[tiab])) OR (fluorin*[tiab] OR fluoro[tiab] OR fluorodeoxyglucose[tiab] OR 18F[tiab] OR 18FDG[tiab] OR F-18DG[tiab]))) OR ((((("ultrasonography"[Subheading] OR "Ultrasonography"[Mesh])) OR ultraso*[tiab]) OR echograph*[tiab]) OR sonograph*[tiab]) OR echotomograph*[tiab]

Embase®

'radiography'/exp OR 'fluoroscopy'/exp OR 'xray'/exp OR 'image subtraction'/exp OR xradiograph*:ab,ti OR xray*:ab,ti OR zonograph*:ab,ti OR fluoroscop*:ab,ti OR arthrograph*:ab,ti OR roentgen*:ab,ti OR rontgen*:ab,ti OR (image NEAR/1 (subtraction OR subtractions)):ab,ti OR 'computer assisted tomography'/exp OR (compute*:ab,ti AND tomograph*:ab,ti) OR 'emission tomography'/exp OR 'tomography'/de OR 'fluorodeoxyglucose f 18'/exp OR 'deoxyglucose'/exp OR pet:ab,ti OR tomograph*:ab,ti OR deoxyglucose:ab,ti OR 2deoxyglucose:ab,ti OR fluorin*:ab,ti OR fluoro:ab,ti OR fluorodeoxyglucose:ab,ti OR 18f:ab,ti OR 18fdg:ab,ti OR f18dg:ab,ti OR 'radioisotope diagnosis'/exp OR'radiopharmaceutical agent'/exp OR 'radioisotope'/exp OR scintigraph*:ab,ti OR scintiphotograph*:ab,ti OR radionuclid*:ab,ti OR radioisotop*:ab,ti OR indium:ab,ti OR in111:ab,ti OR 111in*:ab,ti OR 99mtc:ab,ti OR 99m:ab,ti OR 'gamma camera image':ab,ti OR 'gamma camera images':ab,ti OR 'gamma camera imaging':ab,ti OR ultraso*:ab,ti OR echograph*:ab,ti OR sonograph*:ab,ti OR echotomograph*:ab,ti OR 'echography'/exp OR 'nuclear magnetic resonance imaging'/exp OR (magnetic AND resonance:ab,ti AND (image:ab,ti OR images:ab,ti OR imaging:ab,ti) OR zeugmatograph*:ab,ti OR mri*:ab,ti OR nmr*:ab,ti OR magnetization*:ab,ti OR magnetisation*:ab,ti OR fmri*:ab,ti OR mr AND imag*:ab,ti) AND ('knee prostheses' OR 'joint prostheses' OR 'prostheses' OR 'implantation' OR 'knee arthroplasties' OR prosthe*:ab,ti OR implant*:ab,ti OR arthroplast*:ab,ti OR replac*:ab,ti OR 'metal on metal':ab,ti) AND (knee OR tibia OR 'femur'/exp OR knee:ab,ti OR femoral:ab,ti OR tibia*:ab,ti OR femur*:ab,ti) AND ('infection'/exp OR 'biofilm'/exp OR 'bacterium adherence'/exp OR 'adhesin'/exp OR (infect*:ab,ti OR sepsi*:ab,ti OR septi*:ab,ti OR bacterie*:ab,ti OR bacteria*:ab,ti OR biofilm*:ab,ti OR bacterem*:ab,ti AND orbacteraem*:ab,ti) OR inflammat*:ab,ti)
