# Supplementary material for: What is the Accuracy of Nuclear Imaging in the Assessment of Periprosthetic Knee Infection? A Meta-analysis
Source: Clin Orthop Relat Res. 2017 Jan 3;475(5):1395–410. doi: 10.1007/s11999-016-5218-0 (PMC5384926; doi:10.1007/s11999-016-5218-0)
Supplement: Supplementary file 2 — Supplementary material 2 (DOC 72 kb) [file 11999_2016_5218_MOESM2_ESM.doc]

Appendix 2. PRISMA 2009 flow diagram

Studies Included in Quantitative Synthesis (meta-analysis)

(n = 23)

Studies Included in Qualitative Synthesis

(n = 23)

Full-text Articles Assessed for Eligibility (n = 139)

Records Excluded

(n = 4500 (title)/123 (abstract)

Records Screened

(n = 4639 (title)/262 (abstract)

Records After Duplicates Removed

(n = 4639)

Additional Records Identified Through Other Sources (n = 0)

Identification

Eligibility

Included

Screening

**Records Identified Through Database Searching**

(n = 6572)

Full-text Articles Excluded, With Reasons

(n = 116)
